# Synthesis and Spectrophotometric Analysis of Microcapsules Containing Immortelle Essential Oil

**DOI:** 10.3390/molecules26082390

**Published:** 2021-04-20

**Authors:** Iva Brlek, Anja Ludaš, Ana Sutlović

**Affiliations:** 1 Department of Textile Chemistry and Ecology, Faculty of Textile Technology, University of Zagreb, 10000 Zagreb, Croatia; iva.brlek@ttf.hr (I.B.); ana.sutlovic@ttf.hr (A.S.); 2 Department of Materials, Fibres and Textile Testing, Faculty of Textile Technology, University of Zagreb, 10000 Zagreb, Croatia

**Keywords:** microcapsules, immortelle oil, UV spectroscopy, FE-SEM

## Abstract

In this study, microcapsules were prepared by solvent evaporation technique using ethyl cellulose component as wall and essential oil as core material. The synthesis of microcapsules was carried out using different oil masses. The analysis of the microcapsules was carried out using field emission scanning electron microscope (FE-SEM) and UV spectrophotometric analysis using absorption spectrophotometer. The obtained results confirm the regular spherical shape and size of the synthesized microcapsules. The qualitative and quantitative spectrophotometric analysis of the microencapsulated immortelle oil was measured at the wavelength of 265 nm. The calibration diagram was used to calculate the unknown concentrations of the microencapsulated oil. The obtained results confirm the application of the presented method as relevant for the possible determination of microencapsulated oil on textile materials.

## 1. Introduction

In the last 10 years, microencapsulation has been intensively researched for the medical, bio-medical, agricultural, food, cosmetic, and textile industries [1,2,3,4,5]. Produced micro/nano particles has great contribution to science and economy [2,3,4,5]. Microencapsulation is a process where a functional barrier is established between the core and the wall material to avoid chemical and physical reactions and to preserve the biological, functional and physicochemical properties of the core material [6,7]. It is also a process in which microscopic particles of solids or droplets of liquids (or gasses) are confined in an inert shell, which in turn isolates and protects them from the external environment [8]. Numerous techniques for microencapsulation are available depending on the nature of the encapsulated substance and on the type of polymer used [9,10]. A widely used method for the encapsulation of water insoluble substances within water insoluble polymers is the solvent removal method [2,11,12,13,14,15]. Generally, the resulting product of the microencapsulation process is called a “microcapsule”. Such capsules are micrometer (1 μm) size and have a spherical or irregular shape. Microcapsules can be divided into two parts: The core and the wall. The core (the intrinsic part) contains the active ingredient, while the wall (the extrinsic part) protects the core permanently or temporarily from the external atmosphere [8,16,17]. The microcapsules produced according to the invention can have different broad applications depending on the exact nature of the substance contained therein, as well as on the re-release properties of the substance, which is controlled by the wall-forming material that imparts the properties of the wall of the microcapsules. One example of use is in dermal formulations where substance is dermally active. As will be seen, the invention is not limited by the intended use of the microcapsules or the type of substance encapsulated therein [11,18,19]. 

This research is based on previous successful research by authors in the field of α-tocopherol encapsulating using solvent removal method [20]. Microcapsules has spherical shape, smooth surface and that are important for controlled release and for the chemical stability of the core material [11,20]. So the aim of this study was to microencapsulate, using vapor evaporation technique, and characterize the essential oil of immortelle (lat. *Helichrysum italicum*) in a microcapsule of ethyl cellulose because the major advantage of using microencapsulation technology is its ability to protect the active ingredients from hazardous environments, i.e., oxidization, heat, acidity, alkalinity, moisture, or evaporation [15,21,22,23,24,25]. There are many previous reports of essential oil encapsulation and some of them are based on: How to control the size distribution of capsules, establish that the essential oil was entrapped within the microcapsules rather than being adsorbed onto the surface, the influence of weight ratio of core and wall material, control the main variables (the sample-flow transfer rate, drying air-flow, and temperature) to obtain the best powder recovery and oil content [14,15,22,23,24,25,26,27]. 

There are numerous scientific papers about the essential oils’ encapsulation; however, there is a major gap in encapsulated immortelle oil research. Other major gap is spectrophotometric analysis of immortelle oil. Generally, the UV/Vis spectrophotometric method has been reported by many researchers as the preferred method of essential oils determination because of its relatively low cost, rapidity, high accuracy, and reproducibility [15,25].

The essential oil of immortelle was chosen as the active substance, i.e., the core material for encapsulation. Flowers and leaves are the most commonly used parts in the treatment of health disorders such as allergies, colds, coughs, skin, liver and gallbladder disorders, inflammations, infections, and insomnia. Immortelle is a medicinal plant with promising pharmacological activities. However, most of its traditionally claimed uses have not yet been scientifically proven. Clinical studies are needed to further confirm these data and promote immortelle as an important tool in the treatment of various diseases [28,29,30]. Mediterranean essential oils are characterized by a high content of α-pinene (22%) and appreciable amounts of γ-curcumin (10%), β-selin (6%), nerylacetate (6%), and β-caryophyllene (5%) [31,32]. The proportion of each component in the oil depends on the location of the plant [33]. According to literature, pinene has an absorption peak at 265 nm [34], which is confirmed in this work on qualitative and quantitative UV spectrophotometric analysis of immortelle essential oil in ethyl cellulose microcapsule. Study about applying microcapsules with immortelle oil on textile are already researched by authors [35].

## 2. Experimental

### 2.1. Chemicals

The essential oil of immortelle (EO), supplied by the Croatian company Irex Aroma d.o.o., was used as the active ingredient for the synthesis of the microcapsules. Ethyl cellulose (EC) was used as a wall component in the production of the microcapsules. It was purchased from Sigma-Aldrich, Austria (viscosity 4 cP, 5% in toluene/ethanol 80:20, labelling level: 48% etoxyl). Other chemicals used for the synthesis of the microcapsules were: Ethyl acetate (EA) supplied by Prolabo, as solvent and anionic surfactant, sodium dodecyl sulphate (SDS) from Fluka. The pH in the synthesis was adjusted with citric acid from Prolabo. 

### 2.2. Synthesis of Microcapsules

Ethyl cellulose (EC) microcapsules (MK) were prepared by the phase separation method, in aqueous and organic phase, according to the process as described in Patent No.: US 6932984 B1 [11]. 

Preparation of EC microcapsules in steps:

1. Dissolving or dispersing the substance (immortelle EO) together with a wall-forming material (ethyl cellulose, m = 0.6 g) in an organic solvent (ethyl acetate, V = 15 mL) of the type partially miscible with water to form an organic solution or dispersion;
2. Mixing the organic solution or dispersion with an aqueous solution, the aqueous solution being saturated with the organic solvent (EA, V = 10 mL) dissolved in 100 mL of distilled water and containing an emulsifier (SDS, m = 1 g) to form an emulsion (the pH of the aqueous phase has been adjusted to pH 3 with citric acid to prevent hydrolysis of EA);
3. By mixing the aqueous phase on a magnetic stirrer, the organic phase is gradually added;
4. Adding an excess amount of water (200 mL) to initiate the extraction of the organic solvent from the emulsion (10 min mixing on a magnetic stirrer);
5. Mixing the emulsion long enough to allow formation of microcapsules in the mixture (centrifugation at 2000 rpm for 5 min); and
6. Further removal of the residual organic solvent and formed microcapsules by filtration.

### 2.3. Optimizing of Immortelle Essential Oil Mass in a Microcapsule 

The synthesis of microcapsules was carried out using different oil masses (A = 0.15 g, B = 0.20 g, C = 0.30 g) and microcapsules without oil. Gravimetric analysis was performed on the residues on the philter paper after synthesis.

### 2.4. Morphology Characterization of EC Microcapsules

Surface characterization of the synthesized microcapsules was performed using Field Emission Scanning Electron microscope (MIRA\FE-SEM, Tescan, Czech Republic) operating at an accelerating voltage of 10.00 kV and magnification of 4×–1,000,000×. Prior to analysis, the samples were sputter coated with a chromium alloy (5 min). 

### 2.5. Qualitative and Quantitative UV Spectrophotometric Analysis

Qualitative and quantitative UV spectrophotometric analysis was performed using Spectrophotometer Cary 50 Solascreen (Varian Inc, USA), located in Department of Textile Chemistry and Ecology. The main advantage of UV spectrophotometric method is the ability to being use as easy, safe, rapid, and inexpensive preliminary method [15,25]. Absorption spectrophotometer has working range of 190–850 nm.

Spectrophotometric analysis of essential oil and synthesized microcapsules with three different oil masses (A = 0.15 g, B = 0.20 g, C = 0.30 g) and microcapsules without oil at 265 nm was performed. A calibration curve was prepared for quantitative analysis. To calculate the utilization of immortelle EO in the synthesis of microcapsules (B), this type of synthesis was repeated (B-2). Just before the end of the synthesis (after centrifugation), an aliquot of 0.5 mL was taken from the upper part of the solution and dissolved in 10 mL of hexane. The absorbance at a wavelength of 265 nm was measured using an absorption spectrophotometer. An unknown concentration of immortelle EO, which was not fully synthesized into a microcapsule, was calculated using the equation of a spectrophotometric calibration curve. Comparison of the concentrations of immortelle EO in the microcapsules (B-2) and in the residual solution resulted in utilization according to Equation (1).

m_r_ (%) = (m_r_/m_0_)·100 (1)

where:

m_r_ (%)—percentage of residue MK

m_0_—mass of synthesized MK

m_r_—mass of residue MK.

## 3. Results and Discussion

### 3.1. Synthesis of EC Microcapsules

Table 1 shows the masses of oil in the synthesis of EC microcapsules. Gravimetric analysis was performed on the residues on the philter paper after synthesis.

It is obvious that some of the microcapsules are lost in the final step of synthesis. The greatest loss of microcapsules (17.5%) occurs during synthesis C, which is due to the highest mass of oil. An increase in the residual mass of microcapsules is observed with an increase in the initial mass of immortelle EO. This observation is consistent with the observed consistency of the mixture, in which more immortelle EO is present.

### 3.2. Morphology Analysis of Synthesised Microcapsules with SEM

**Figure 1:**
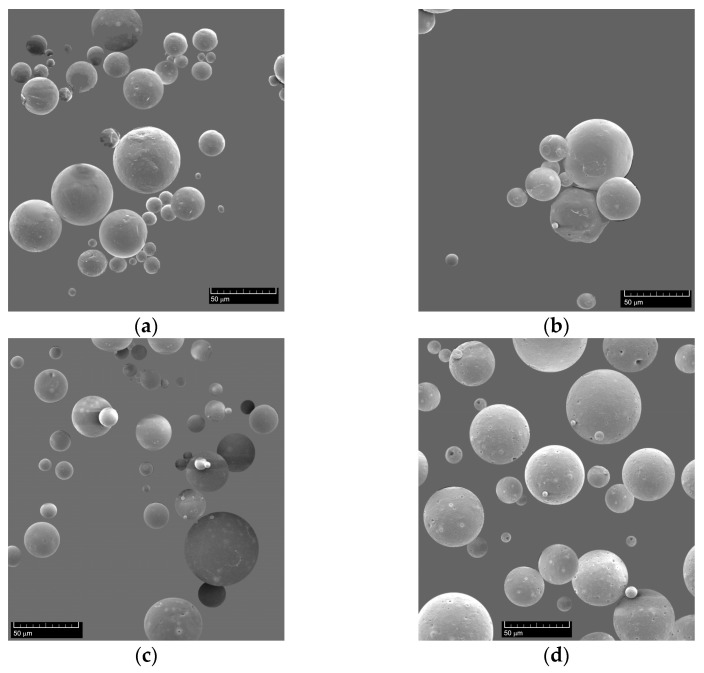
SEM images of ethyl cellulose microcapsules with three different oil masses and empty microcapsules, (**a**) A (m1 = 0.15 g), (**b**) B (m2 = 0.20 g), (**c**) C (m3 = 0.30 g), and (**d**) 0 (microcapsules without oil), magnification 1000x.

SEM Figures (Figure 1) confirmed the quality of the synthesized ethyl cellulose microcapsules, which were characterized by their regular spherical shape. The microcapsules were in the size range of 10–50 μm with an average diameter of 30 μm.

Microcapsules synthesized with 0.200 g Immortelle EO are the best (B). They have intense odor and less losses, the microcapsules can be easily removed from the filter paper.

### 3.3. Spectrophotometric Analysis of Immortelle EO

#### 3.3.1. Qualitative Analysis of Microcapsules

**Figure 2:**
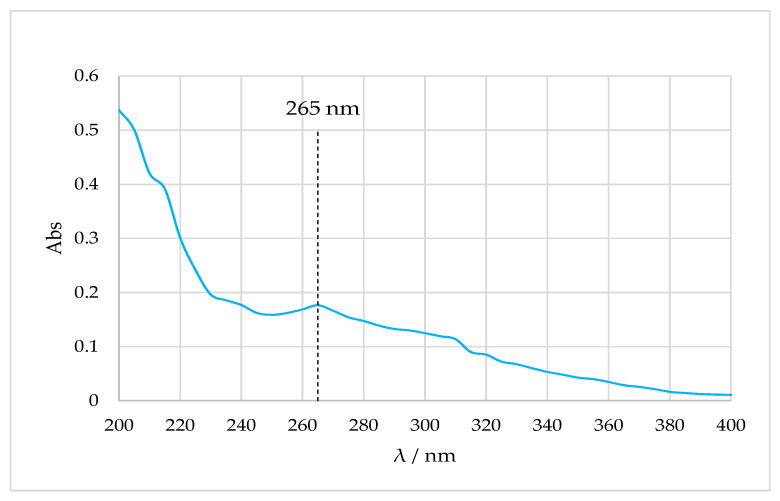
Absorption curve of immortelle EO, range 400–200 nm.

Qualitative analysis of immortelle EO was done by UV spectrophotometry with maximum absorption at the peak at 265 nm (Figure 2) in accordance with the literature [15,34].

#### 3.3.2. Quantitative Analysis of Microcapsules

**Figure 3:**
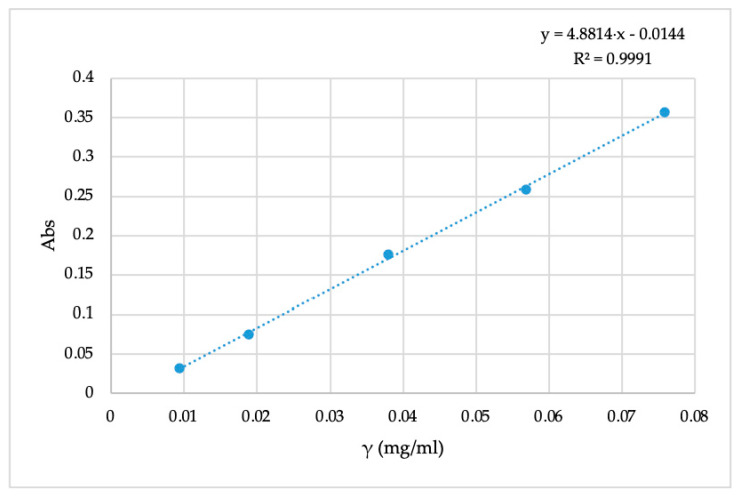
The calibration diagram.

The UV spectrophotometer Cary 50 Solascreen was used to measure the absorbance of various concentrations of immortelle EO at different wavelengths (200–400 nm). Table 2 shows the absorbance values and concentrations of the oil for the calibration diagram. Using the concentrations and measured absorbance of 5 different solutions of immortelle EO, a calibration diagram was constructed in Figure 3. The equation of a spectrophotometric calibration curve corresponding to y = 4.8814x − 0.0144, where R^2^ = 0.9991, shows the high linear correlation between immortelle EO concentration and peak area at 265 nm.

**Figure 4:**
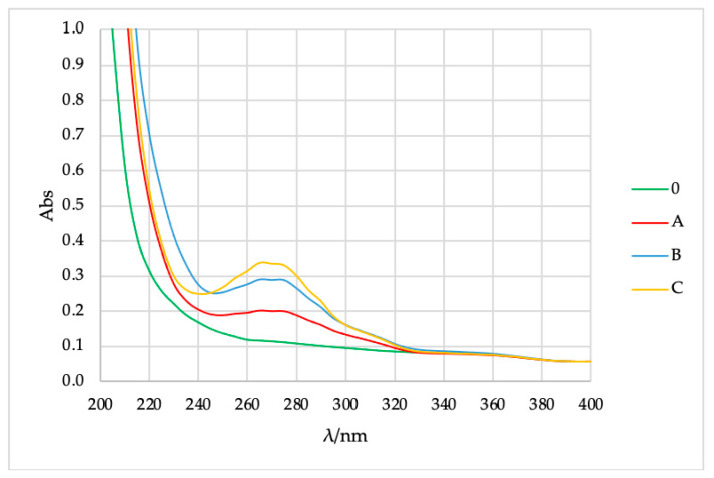
The absorbance of immortelle EO in microcapsules, varying amount of oil.

Then, 1 mg of the synthesized microcapsules, different amounts of immortelle EO (0 = 0.0 g, A = 0.15 g, B = 0.20 g, C = 0.30 g) were dissolved in 10 mL of hexane (stirring for 5 min) and the absorbances were measured by spectrophotometer. From Figure 4 it can be seen that all three microcapsules (A, B, and C) have maximum absorbance at 265 nm. Microcapsules without oil (0) have no absorption maximum. This means that the presence of oil can be detected in the previous solutions of the microcapsules.

From the straight line equation (y= 4.8814x − 0.0144, R^2^= 0.9991) and the measured absorbance, the unknown concentration of immortelle EO in microcapsules was calculated as shown in Table 3.

#### 3.3.3. Utilization

Table 4 shows the amount of utilization of immortelle EO in the synthesis of microcapsules (B-2). The absorbance at a wavelength of 265 nm was measured on an absorption spectrophotometer, and the unknown concentration of immortelle EO, which was not fully synthesized into a microcapsule, was taken after centrifuge and calculated using a calibration diagram.

From the concentration of microcapsules B-2 (γ = 0.0627 mg/mL) and the concentration of the residual solution (γ = 0.0130 mg/mL) the utilization of high 82.83% for immortelle EO was obtained. This means that only 17.17% of immortelle EO has not been synthesized.

## 4. Conclusions

The results obtained in this research confirmed influence of immortelle EO mass on EC microcapsules properties. EC microcapsules with immortelle EO with four different mass of oil were synthetized. Microcapsules synthesized with 0.20 g of immortelle EO represent the best choice because they have less losses in synthesis, the amount of synthetized microcapsules are acceptable and subjectively they have expected intense smell.

SEM morphology analyses confirmed that all microcapsules have regular spherical shape and the mass of oil used in synthesis does not have influence on microcapsules size.

Based on the results, it can be concluded that spectrophotometric investigation of immortelle EO and immortelle EO in microcapsules is appropriate for qualitative and quantitative analysis. In summary, it can be concluded that this method is suitable and relevant for possible determination of microencapsulated immortelle oil on textile materials.

## Figures and Tables

**Table 1:** Synthesis of microcapsules with the corresponding results of gravimetric analysis.

Type of Synthesis	m (EO)/g	m_0_ (MK)/g	m_r_ (MK Residue on Filter Paper)/g	m_r_ (MK Residue on Filter Paper)/%
**0**	/	0.130	0.015	11.5
**A**	0.15	0.429	0.042	9.8
**B**	0.20	0.389	0.040	10.3
**C**	0.30	0.464	0.081	17.5

**Table 2:** Concentrations and the measured absorbance of 5 different solutions of immortelle EO at 265 nm.

No.	Absorbance	γ/mg/mL
1	0.0324	0.01
2	0.0752	0.02
3	0.1764	0.04
4	0.2589	0.06
5	0.3569	0.08

**Table 3:** The calculated concentration of microcapsules and measured absorbance.

Synthesis	Mass of Immortelle EO in Microcapsules, m/g	Absorbance	γ/mg/mL
**0**	0	0	0
**A**	0.15	0.2023	0.0444
**B**	0.20	0.2915	0.0627
**C**	0.30	0.3376	0.0721

**Table 4:** Utilization of immortelle EO in microcapsules synthesis (B-2).

Immortelle EO	A	γ/mg/mL	Utilization/%
**In microcapsules (B-2)**	0.29145	0.0627	82.83
**In residual solution**	0.04902	0.0130	

## Data Availability

The data presented in this study are available on request from the corresponding author.

## Sample Availability

Samples of the compounds are available from the authors.
